# Bioremediation of environments contaminated with mercury. Present and perspectives

**DOI:** 10.1007/s11274-023-03686-1

**Published:** 2023-07-13

**Authors:** Daniel González-Reguero, Marina Robas-Mora, Agustín Probanza Lobo, Pedro Antonio Jiménez Gómez

**Affiliations:** Department of Pharmaceutical Science and Health, CEU San Pablo University, Montepríncipe Campus, Ctra. Boadilla del Monte Km 5.300, 28668 Boadilla del Monte, Madrid, Spain

**Keywords:** Mercury1, Biorremediation2, Environment3, Microbial community4

## Abstract

Mercury is a highly toxic heavy metal whose emission sources can be both natural and the result of anthropic activity. Its polluting action on soils, and its ability to spread through the atmosphere and aquatic environments, constitutes a threat to human and environmental health; both for its bioaccumulation capacity and for biomagnification through the trophic chain. For this reason, there is a growing scientific and social interest in the reduction of this heavy metal in ecosystems. Bioremediation based on the use of microorganisms and/or plants is postulated as a sustainable alternative to traditional physicochemical methods. The main strategies used for this purpose (individually or in combination) are the volatilization of the contaminant, biosorption, phytoextraction and phytoremediation. All these tools are based on taking advantage of the natural and evolutionary capacity that different organisms have developed to adapt to the presence of various pollutants in the environment. Based on the consulted bibliography, these bioremediation methodologies focus on the use of microorganisms (freely or associated with plants) have been successfully applied in different ecosystems, postulating themselves as a respectful alternative for the future for the recovery of degraded environments. For these reasons there is a growing interest in the scientific community to design and use new techniques in a “One Health” context, which allow interpreting the positive impact of bioremediation. In this sense, the universalization of Omics techniques has allowed to abound in the knowledge of new bacterial taxa, and their biotechnological application. This study pretends to cover the present knowledge about mercury bioremediation techniques. In the same way, some new techniques and perspectives are presented in order to expand the frontiers of future research.

## Mercury as a pollutant

Mercury (Hg) is one of the most toxic heavy metals. Pollution by this element is a serious environmental problem, even at low concentrations, which affects all systems: soil, water and air (Munthe et al. 2019; Ballabio et al. 2021; Attwaters 2023).

Most of the environmental Hg is in the form of inorganic and organomercuric salts, except for atmospheric mercury. The most prevalent species in the environment are mercuric salts, such as HgCl_2_, Hg(OH), and HgS. In addition, CH_3_HgCl, and CH_3_HgOH are the main organomercurial compounds that together with other organic compounds are found in small fractions (Al-Sulaiti et al. 2022). The organomercurial compounds mentioned are compounds derived from methylmercury (MeHg or [CH^3^ Hg]^+^), one of the most dangerous Hg species, due to its high capacity to bioaccumulate in the tissues of organisms (Munthe et al. 2019; Gallorini and Loizeau 2021; Li et al. 2022b).

The most relevant Hg emitting sources are those of natural, anthropogenic and re-emission origin (Panagos et al. 2021; Sonke et al. 2023). It is estimated that since the beginning of the industrial revolution (AMAP/UNEP 2013), the amount of global atmospheric Hg has increased 10-fold and that throughout the post-industrial era to the present, the amount of Hg accumulated in soils and sediments has increased 3–10 times (Munthe et al. 2019).

The most important anthropogenic sources of Hg pollution are urban and industrial discharges, agricultural materials, mining, combustion, which emit from 2000 to 2200 tons annually, with the main source being the burning of fossil fuels and waste incineration (Munthe et al. 2019; Ballabio et al. 2021; Singh et al. 2023c; Zhang et al. 2023). These emissions come mainly from the metallurgical industry of non-ferrous materials, the main one being the Zn industry, followed by the large-scale production of Au, Cu and Al (Munthe et al. 2019; Yuwono et al. 2023). Finally, as a polluting source, there are reemissions, which are defined as Hg emissions derived from natural and anthropogenic past deposits. Under the right conditions, Hg deposits at the Earth’s surface can be suspended back into the atmosphere by various transport mechanisms. Annual Hg re-emission is estimated to be between 4,000t and 6,300t per year (Munthe et al. 2019; Ballabio et al. 2021). Most of this re-emitted Hg accumulates back in the soil.

## Mercury-contaminated environments

**Fig. 1:**
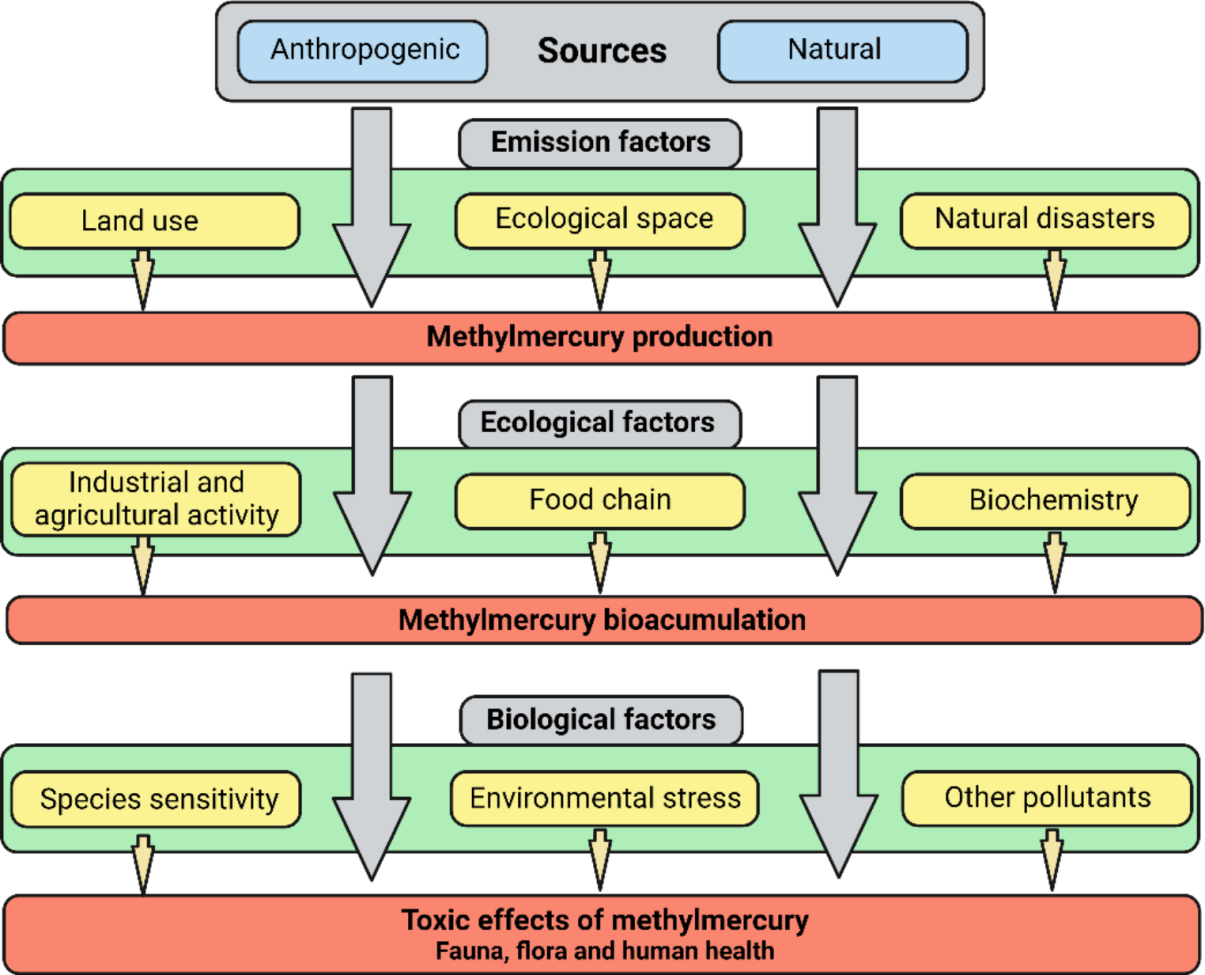
Flow of Hg in the environment. Own elaboration

Numerous ecosystems and environments contaminated with Hg are known, especially in regions with a high level of industrial activity and cities with large population volumes (Munthe et al. 2019) (Fig. 1).

The natural environments in which higher concentrations of Hg are detected are those indicated below:

### Atmosphere

Elemental Hg (Hg^0^) and divalent Hg (Hg^2+^) are the majority species found in the atmosphere. The latter species is composed of gaseous divalent Hg (Hg^2+^) and divalent Hg particles (Hg^ρ^) (Munthe et al. 2019; Dastoor et al. 2022; Yuan et al. 2023). Atmospheric Hg can be deposited in aquatic and terrestrial ecosystems through sedimentation and rainfall. The main specie of Hg that is deposited is the gaseous divalent Hg, coming mainly from anthropogenic and reemission sources; being the atmosphere the main recipient that collects and distributes them globally. In this way, atmospheric accumulation contributes significantly to the transport and discharge of Hg to multiple environments. Hg^0^ present in the atmosphere can be deposited in the medium after conversion to Hg^2+^ after its reduction by ozone (Saiz-Lopez et al. 2022).

### Aquatic systems

Hg exhibits high bioavailability in aquatic systems, since under appropriate biochemical conditions, inorganic Hg is converted to MeHg by microorganisms (Gallorini and Loizeau 2021; Yue et al. 2023). It is known that the main source of biotransformation of Hg to MeHg in aquatic systems occurs in bed sediments as well as microorganisms along the water column (Gallorini and Loizeau 2021; Li et al. 2022b). This fact facilitates its accumulation in marine organisms, which can produce a high concentration of Hg in themselves (Liu et al. 2020; Scott and Black 2020; Li et al. 2022b). This problem is well described in some environments, such as river mouths, large cities, and the South China Sea (Xiang et al. 2018; Bernalte et al. 2020). The different aquatic systems, and mainly the oceans, act as Hg sinks, with the consequent ease of Hg biotransformation.

### Soil

More than 90% of the emitted Hg ends up back in terrestrial ecosystems, with soil being the largest deposit of this metal (Ballabio et al. 2021; Yuwono et al. 2023). About 1–3% of the Hg present in soils is MeHg and the remaining percentage corresponds to different complexes, maintaining a small part as Hg^0^ (O’Connor et al. 2019). The capacity of the soil to accumulate Hg arouses scientific interest due to the easy transmissibility contaminants to organisms that develop in it. In this way it can be incorporated into the trophic chain, through its bioaccumulation in plants for human consumption or livestock. In turn, these compounds can move from soil deposits to the aquatic environment (Gębka et al. 2020; Yuwono et al. 2023), polluting the media, on a large scale. Likewise, the highest levels of Hg in soil have been found near urban centers with high industrial activity, as well as mining areas (Ballabio et al. 2021; Panagos et al. 2021). Hg forms, under natural conditions, primary complexes with Cl-, OH-, S^2−^ and with organic compounds containing sulfur functional groups. Organic compounds have also been seen to be the dominant factor in Hg mobilization (Al-Sulaiti et al. 2022).

## Effects of mercury pollution on systems and organisms

### Hg in soil microbial communities

Hg, as a heavy metal and pollutant, exerts a strong biological and environmental pressure that affects the structure of microbial communities and their diversity (Mariano et al. 2020; Hu et al. 2023). The *mer* operon is one of the best-known bacterial defense systems against Hg (Manoj et al. 2020; Yadav et al. 2023). *merA* is the central gene of this operon, which codes for a mercuroreductase enzyme whose function is to catalyze the reduction of volatile Hg^2+^ to Hg^0^ (Harsonowati et al. 2023). These resistance genes are usually included in plasmids and other mobile gene elements, very ubiquitous in ecosystems. Two types of *mer* operon are known, capable of providing bacteria with Hg resistance (Naguib et al. 2019): (i) reduced spectrum operon which confers resistance only against inorganic Hg; (ii) broad-spectrum operon that, in addition to inorganic Hg resistance genes, include additional *mer* genes that confer organormercuric species resistance.

**Fig. 2:**
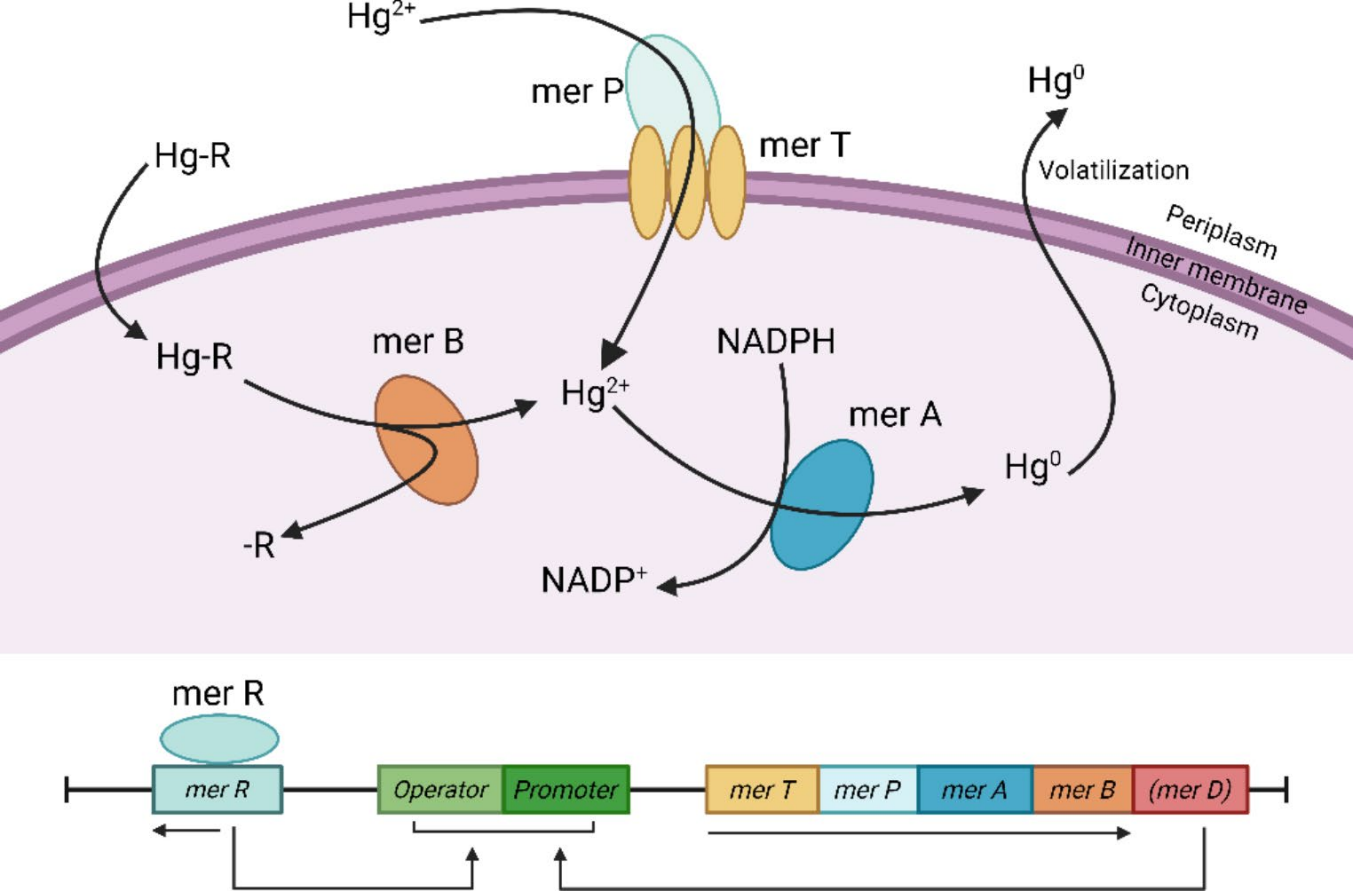
*mer* operon schematization. *merA*: Hg reductase; *merB*: organomercuric lyase. *merT*: Hg transporter; *merP*: periplasmic Hg transport; *merR*: operon regulator; *merD*: involved in regulation. Own elaboration

The biochemical process of inorganic Hg resistance is very similar between different bacterial species. In the case of bacteria with the reduced spectrum *mer* operon, there is a conversion of Hg^2+^ to Hg ^0^ mediated by a reductase enzyme produced by the *merA* gene, induced by Hg^2+^. This enzyme uses NADPH as an electron source. Organomercuric compounds resistance biochemical process varies according to the bacterial species. After the transport process, the bond between Hg and carbon is digested by a lyase enzyme, encoded in the *merB* gene, releasing Hg^2+^. The Hg cation is subsequently transformed into Hg^0^ by a mercuroreductase encoded by *merA* (Kumari et al. 2020) (Fig. 2).

The appearance of the contaminant in the soil favors the selection of those strains that present a greater tolerance to acquire resistance genetic elements that may be present in the environment. (Hall et al. 2020; Li et al. 2022a). In turn, Hg affects the composition of bacterial communities, decreasing their diversity (Mariano et al. 2020; Zheng et al. 2022; Hu et al. 2023). Likewise, the natural selection of tolerance to heavy metals can be linked, by a phenomenon of co-selection, to resistance to other compounds such as antibiotics (Robas et al. 2021b; Karnachuk et al. 2023; Tran et al. 2023), as well as the horizontal transfer of these resistances (Robas et al. 2021b; Li et al. 2022a; Kothari et al. 2023).

### Hg in crops

Inorganic Hg can be incorporated and sequestered from soils into plant tissues by both stomal and non-stomal absorption (Zhou et al. 2021; Hussain et al. 2023; Singh et al. 2023a). The association of this heavy metal with functional groups of organic matter and various root exudates capable of retaining Hg in the soil (Eagles-Smith et al. 2018; Du et al. 2019; Hussain et al. 2023) favor the retention of Hg in ecosystems. The relationship between soils and plant activity is one of the most important environmental markers of soil inorganic Hg variation (Munthe et al. 2019; Ballabio et al. 2021).

### Hg in the food chain

**Fig. 3:**
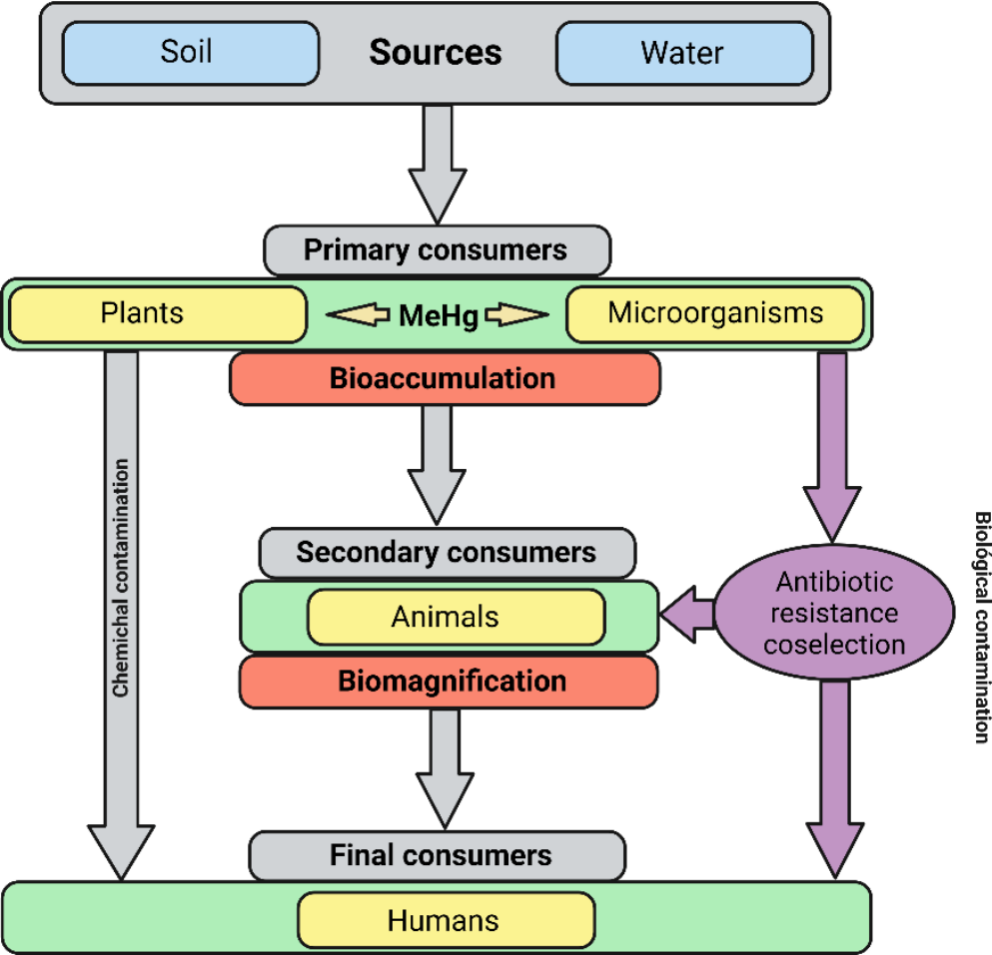
Flow of Hg in the food chain (chemical contamination) and potential source of antibiotic resistance (biological contamination). Own elaboration

The presence of Hg in ecosystems allows its incorporation into the food chain (Li et al. 2022b; Basu et al. 2023; Han et al. 2023; Moslemi-Aqdam et al. 2023), negatively affecting, in the One health context, the health of ecosystems, especially the species that are at the highest levels of the chain (Li et al. 2022b). Most of the forms that Hg can take in nature are highly toxic to all species, even at low concentrations (Gil-Hernández et al. 2020; Marumoto et al. 2020; Basu et al. 2023). The processes known as bioaccumulation (accumulation of toxic substances in tissues) and biomagnification (increased accumulation of toxicants due to predation or consumption of other contaminated organisms), ultimately affect human health (Basu et al. 2023; Han et al. 2023; Moslemi-Aqdam et al. 2023) (Fig. 3).

The levels of Hg can be found in terrestrial vertebrates intended for human consumption are very low (Emami et al. 2023; Nava et al. 2023). However, numerous authors collect evidence of a high concentration of Hg in fish (Alemayehu et al. 2023; Frías-Espericueta et al. 2023). Despite the high concentration of Hg that can be found in the oceans, studies such as those conducted by Li et al. (2022b), show how water from rivers and lakes have a higher concentration of Hg than open waters and coasts. Hg and MeHg biomagnify along the food chain from phytoplankton to zooplankton to higher organisms (Frías-Espericueta et al. 2023; Han et al. 2023; Yue et al. 2023).

To address this environmental and health problem, the United Nations Minamata Convention on the Reduction of Hg Emissions and Use (AMAP/UNEP 2013) has developed regulations for the control of emissions to air, water, or waste and products under Federal environmental statutes, with the acts of *“clean air, clean water and the recovery and conservation of natural resources”* (Aldy et al. 2020).

Chronic exposure to this pollutant, especially through the consumption of seafood, can cause various neurological alterations, reproductive and immunological conditions and premature death (Gil-Hernández et al. 2020; Marumoto et al. 2020; Basu et al. 2023; Mallongi et al. 2023), especially affecting embryos and people suffering sustained exposure over time (Lee et al. 2023). Likewise, the case of Hg disease in humans that occurred in Minamata (Japan) between 1932 and 1968 is also well analyzed. During this period, an acetic acid factory dumped waste liquids with a high concentration of MeHg into Minamata Bay, where a large population subsisted on fishing for self-consumption. Even today, it has been observed that in populations engaged in subsistence fishing, between 1.5 and 17 out of every thousand children have cognitive disorders (mild mental retardation) due to the consumption of contaminated fish (Malagon-Rojas and Sonia 2018; Lin et al. 2023).

### Bioremediation

The potential capacity of microbial communities and their use for the improvement of plant production (Valle-Romero et al. 2023; Vlajkov et al. 2023; Nagrale et al. 2023), as well as the use of soil plant processing capacity to reduce the presence of a certain pollutant (Chandel et al. 2023; Sitarska et al. 2023; Nnaji et al. 2023) are issues that have been addressed from different perspectives. Bioremediation or biotechnological remediation of a contaminated environment is presented as an economical alternative with a lower negative impact on the environment (Daniel et al. 2022). The use of mercurotolerant microorganisms (Mathema et al. 2011) represents a great opportunity for the design and application of effective bioremediation processes (Yadav et al. 2023; Rojas-Solis et al. 2023; Gupta et al. 2023).

**Fig. 4:**
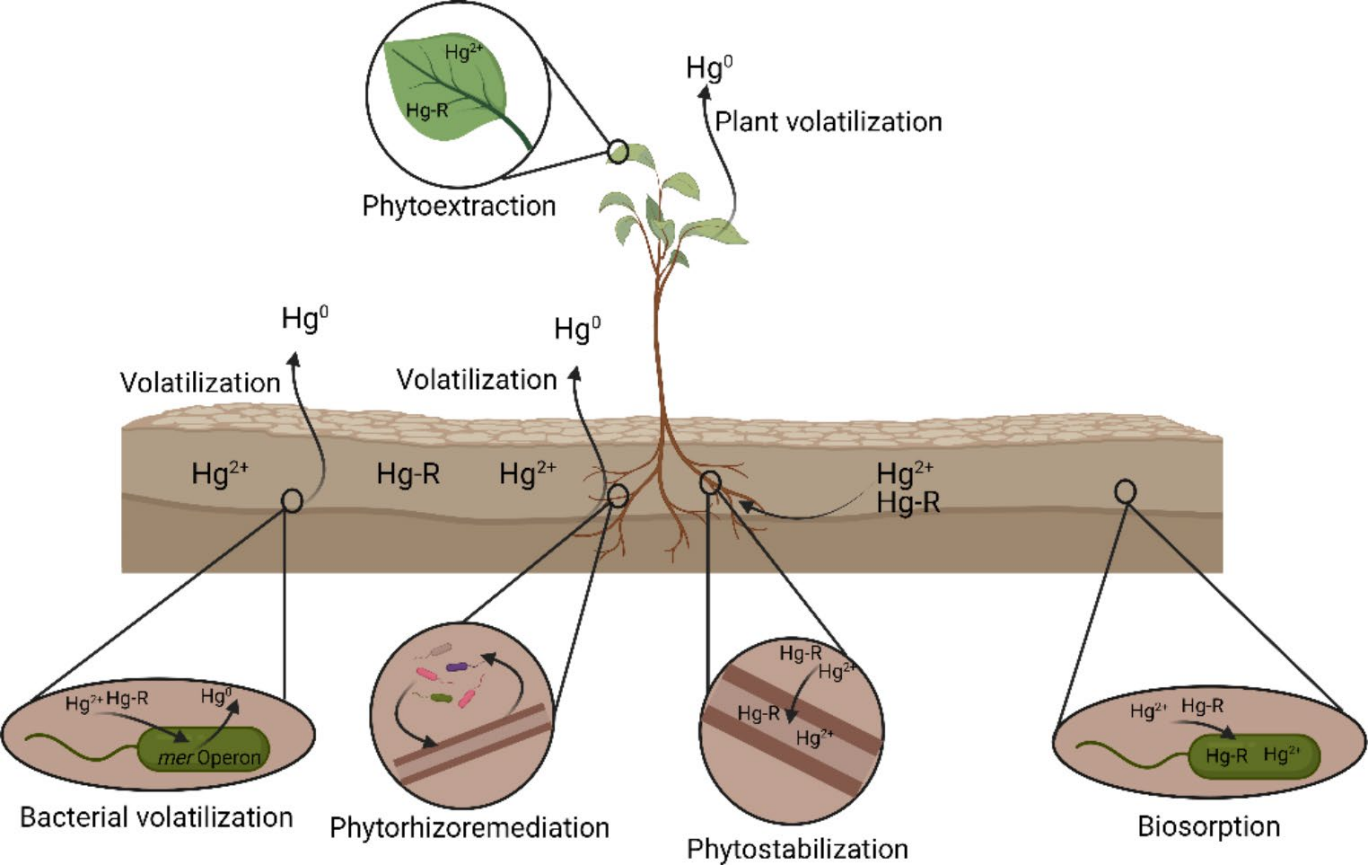
Main mercury bioremediation strategies. Own elaboration

In the literature, a large number of works can be found regarding the biological methods used to remediate Hg with bacteria:

- Volatilization of the contaminant: Bacteria possessing the *mer* operon are able to reduce Hg from Hg^2+^ to volatile Hg^0^ (Tanwer et al. 2022; Yadav et al. 2023; Yao et al. 2023). The *mer* operon codes for a mercuroreductase (*merA*), an organomercuric lyase (*merB*), a periplasmic protein for environmental Hg uptake (*merP*), inner membrane proteins related to Hg^2+^ transport (*merT*, *merC*, *merE*, *merF* and merG) and operon system regulatory proteins and expression (*merR* and *merD*) (Gionfriddo et al. 2020).
- Biosorption of Hg: Defined as the ability of microorganisms to capture heavy metals by increasing their biomass (Vijayaraghavan and Yun 2008; Beveridge and Murray 1980). The heavy metal is retained in the bacterial cell wall, without the need for intracellular bioaccumulation (Jing et al. 2022; Baran et al. 2022; Yao et al. 2023; Zhao et al. 2023).
- Phytoextraction (Yu et al. 2022; Sitarska et al. 2023; Nnaji et al. 2023):. A process by which it is intended to remove heavy metals through the use of plants: (i) Fitoextractors: when the metal accumulates in the aerial part (Wang et al. 2019), and (ii) Phytostabilizers: when accumulation occurs in the root (Dary et al. 2010; Ke et al. 2021).
- Phytoremediation (or phytorhizoremediation): Technique that uses plant-microorganism interaction for the extraction and/or removal of soil contaminants (Raklami et al. 2022; Ruley et al. 2022; Sitarska et al. 2023; Rojas-Solis et al. 2023). This technique has a much more powerful and efficient effect by taking advantage of the synergistic action between the plant and microorganisms (Quiñones et al. 2021; Robas et al. 2021a; Senabio et al. 2023) (Fig. 4).

## New opportunities for old Problems: plant-microorganism interaction

Plants and microorganisms show parallel patterns of heterogeneity because plants release a wide range of organic products (via exudate), which are consumed by soil microorganisms. In this way, it has been postulated that the taxonomic heterogeneity of plants improves plant productivity by making more efficient use of available resources. But this factor has to go hand in hand with microbial activity in the soil and the bioavailability of organic resources.

In the plant-microorganism interaction, two fundamental factors should be considered: (i) to know the way in which the plant selects the rhizospheric microbiota and the way of relating to the microorganism; (ii) understanding microbial functional diversity will help reveal underlying ecological processes. In plant-microorganism collaboration, the benefit of this synergy in soil recovery is diminished in the presence of contaminants (Irfan et al. 2023). In this way, it is shown how the impact of heavy metals alters and reduces the diversity of the soil microbial ecosystem (Mariano et al. 2020; Robas et al. 2021a, c; González et al. 2022).

Some microorganisms have the ability to protect plants against biotic or abiotic agents that compromise their normal development (Robas et al. 2022; Nagrale et al. 2023; El-Sayed et al. 2023), mainly by volatilization and bioadsorption mechanisms (Robas Mora et al. 2022; Yadav et al. 2023; Zhao et al. 2023). Phytoprotection is a very interesting quality with potential agronomic use in contaminated soils that would otherwise take long periods of time to recover.

Some pollutants, such as Hg, induce physiological and metabolic alterations in plants, such as the appearance of ROS (reactive oxygen species) and decreased plant growth (Çavuşoğlu et al. 2022). Likewise, it is known that the use of PGPB minimizes these effects (Pirzadah et al. 2018). One of the main effects that numerous environmental pollutants have on a plant is the increase in oxidative stress and the production of ROS (Çavuşoğlu et al. 2022; Carrasco-Gil et al. 2023; Flores-Cáceres et al. 2023; Magnuson and Sandheinrich 2023). The production of the enzymes superoxide dismutase (SOD), glutathione reductase (GR), catalase (CAT) and ascorbate peroxidase (APX) catalyze the degradation of ROS such as H_2_0_2_, HO-, ^1^O_2_ and the superoxide anion O^−^ _2_. This effect has also been observed in the cellular production of APX and GR enzymes when confronting different plant species with heavy metals (Liu et al. 2018; Azimychetabi et al. 2021). Therefore, enzyme activity is interpreted as a protective response against ROS, whose function is induced by the intracellular presence of Hg (González-Reguero et al. 2022).

## Microorganisms and new tools for bioremediation

### Tools for the characterization and description of new strains. Massive Sequencing (NGS) and Biomercuroremediator Suitability Index (BMRSI)

During the last four decades, the use of microbiological agents (fungi and bacteria) as an alternative to conventional chemicals has prevailed. The commonly used plant growth promoting bacteria (PGPB) species are those belonging to *Bacillus* (Etesami et al. 2023; Liu et al. 2023; Wróbel et al. 2023) and *Pseudomonas* genera (de Andrade et al. 2023; Singh et al. 2023b).

An indirect effect of phytoprotection is the ability of these bacteria to prevent or reduce damage to the plant by the action of certain pathogens (Nagrale et al. 2023; El-Sayed et al. 2023). To do this, the accompanying bacteria can induce resistance mechanisms of the plant itself. PGPBs can perform other beneficial functions against abiotic stress for plants, such as protecting against salinity, drought, or toxic environmental compounds, such as heavy metals (Rajendran and Sundaram 2020; Zerrouk et al. 2020; Danish et al. 2020; Wróbel et al. 2023).

The main activities that allow characterizing a strain as PGPB have been the production of different phytohormones (like 3-indolacetic acid; IAA) and growth promoting activities (Basu et al. 2021; Hsu et al. 2021; Valle-Romero et al. 2023; Nagrale et al. 2023; Gupta et al. 2023). Likewise, it is necessary to know the Hg minimal bactericidal concentration (MBC) as a measure of its mercurotolerant capacity (Mathema et al. 2011). To assess these factors in an integrated wayRobas et al. (2021a); González et al. (2021) propose the use of the BMRSI (Biomercuroremedial Suitability Index):

BMRSI= [IAA (mg.mL^− 1^) + ACCd (1/0) + SID (cm) + PO_4_^3−^ (1/0)] + [MBC Hg (mg.mL^− 1^)]

Where 1 = Presence; 0 = Absence; IAA: Indole-3-acetic acid production; ACCd: ACC deaminase activity; SID: Siderophores production; PO_4_^3−^: Phosphates solubilization capacity; MBC: Hg minimal bactericidal concentration.

The development of genetics and whole genome sequencing (through Next Generation Sequencing (NGS) and Whole Genome Sequencing (WGS) are fundamental tools for the description of new bacterial strains, their taxonomic reassignment (Mora et al. 2022; Shu and Huang 2022) or the molecular study of uncultivable species (Cycil et al. 2020) introducing a new paradigm in the study of microorganisms. Likewise,the use of new bioinformatics tools has allowed the discovery of new genes and molecular mechanisms of residence to metals such as Hg (Douglas et al. 2020; Moon et al. 2020).

### Community study tools. Cenoantibiogram and Omics-

The presence of antibiotics in soils is an inherent fact because soil systems are the habitat of numerous microbial species that naturally produce them. Antibiotics may, at sub-inhibitory concentrations, have different functions such as the activation/deactivation of virulence factors or the regulation of microbial communication systems (Li et al. 2021; Chow et al. 2021; Moori Bakhtiari et al. 2023). The effect of antibiotic resistance in different populations may be due to various factors, including the effect of microbial communication processes and/or ecological competition, such as *quorum-sensing/ quorum-quenching* (Li et al. 2021; Wang et al. 2023), as well as the response to abiotic factors and genetic co-selection with resistance mechanisms to the presence of heavy metals. One of these tools that allows the study of antibiotic resistance of a complex microbial community is the so-called *cenoantibiogram* (Mora et al. 2017). The interpretation of the cenoantibiogram does not seek to characterize all the different mechanisms that explain each of the antibiotic resistances detected but the overall behavior of the soil microbial community compared to the most commonly used antibiotics. This technique is suggested as a possible bioindicator of both the evolution of the edaphic community and the comparison between different communities.

Likewise, there is no doubt that there is a growing interest in the use of techniques such as metagenomics, transcriptomics, proteomics and metabolomics for the study of soil samples and the use of microorganisms in bioremediation processes (Chakdar et al. 2022; Jhariya and Pal 2022; Sharma et al. 2022; López and dos Santos Silva 2023; Sevak et al. 2023). Methods based on the functional analysis of DNA libraries can be a great source for the discovery of new genes for resistance to different pollutants, such as heavy metals (Sharma et al. 2021; Chen et al. 2022). Similarly, transcriptomic and proteomic analysis offers an opportunity for understanding the expression of these genes and the different mechanisms of resistance, their expression and their regulation (Jhariya and Pal 2022; Lata et al. 2023; López and dos Santos Silva 2023; Shyam et al. 2023).

### Future prospects

Bioremediation and the use of biotechnological methods for the removal of environmental pollutants, such as Hg, is proposed as an economical and environmentally friendly alternative. Therefore, the development of new technologies that allow effective environmental remediation is, at present, a field of research of great scientific interest. To this end, it is necessary to develop procedures and techniques that allow the correct taxonomic ascription or classification of new PGPB with potential biotechnological use. In order to successfully employ new microbial species in bioremediation processes, a deeper understanding of the mechanisms that promote microbial activity (such as communication mechanisms mediated by *quorum sensing*) and the metabolism of pollutants under various ecological conditions (through the aforementioned processes of mobilization/immobilization, translocation, transformation, biosorption or bioaccumulation) is needed. “Omics” tools will continue to be key to the discovery of new microbial species, genetic mechanisms of transformation (metagenomics); gene expression in different biological contexts (metatranscriptomics); the synthesis and participation of proteins in new metabolic pathways (metaproteomics) as well as the identification of metabolites not yet described with therapeutic, industrial, or remedial potential (metabolomics).

It is necessary to expand the knowledge of the mechanisms that allow the exosimbiont association or mutualistic interaction between organisms, especially those established between plants and microorganism, which allow an optimization of the use of organisms for the recovery of degraded environments. Finally, obtaining genetically modified microorganisms will allow the development of more effective methods and techniques for the restoration of the balanced conditions of deteriorated ecosystems.

## Data Availability

There are no data associated with this publication.
